# Comparison of Refractive Prediction Errors of Toric Intraocular Lens Formulae

**DOI:** 10.7759/cureus.79679

**Published:** 2025-02-26

**Authors:** Michelle Bai, Keith Ong

**Affiliations:** 1 Department of Ophthalmology, Cairns Hospital, Cairns, AUS; 2 St George and Sutherland Clinical Campuses, School of Clinical Medicine, University of New South Wales, Sydney, AUS; 3 Department of Ophthalmology, Royal North Shore Hospital, Sydney, AUS; 4 Northern Clinical School, University of Sydney, Sydney, AUS; 5 Department of Surgery, Chatswood Private Hospital, Sydney, AUS; 6 Department of Surgery, Sydney Adventist Hospital, Sydney, AUS; 7 Save Sight Institute, University of Sydney, Sydney, AUS

**Keywords:** astigmatism correction, iol power calculation formula, refractive cataract surgery, refractive prediction error, spherical equivalence, toric intraocular lens

## Abstract

Introduction

Toric intraocular lenses (IOLs) are effective in correcting astigmatism; however, with multiple IOL toric formulae available, identifying the most accurate formula is important. The IOLMaster 700 (Carl Zeiss Meditec AG, Jena, Germany) provides two in-built toric IOL formulae, the Barrett TK Toric (BTT) and Haigis Toric (HT), which sometimes differ in astigmatism predictions for a particular IOL power. This study investigates the accuracy of these formulae in predicting postoperative refraction.

Methods

This retrospective study included 52 eyes from 52 patients undergoing uncomplicated cataract surgery with AcrySof IQ Toric IOL implantation (Alcon Laboratories Inc., Fort Worth, Texas, United States). Biometric data, including axial length, anterior chamber depth, lens thickness, and total keratometry, were measured using the IOLMaster 700. Predicted residual spherical equivalence (SE) and astigmatism were calculated using BTT and HT formulae. Postoperative refractive outcomes were assessed at least four weeks post-surgery. Differences in prediction errors in SE and astigmatism were assessed.

Results

The median absolute error in SE prediction was comparable between BTT and HT formulae (0.195 D vs. 0.185 D; p=1.000), with no significant difference in the proportion of eyes achieving absolute prediction errors ≤0.25 D, ≤0.50 D, ≤0.75 D, or ≤1.00 D. For astigmatism, the HT formula demonstrated lower mean absolute errors (MAE: 0.41±0.33 D) and centroid errors (0.18 D @ 70°) compared to BTT (MAE: 0.47±0.36 D; centroid error: 0.32 D @ 76°; p<0.001).

Conclusion

Both BTT and HT formulae exhibit similarly high accuracy in predicting postoperative SE. HT performed statistically better for astigmatism prediction; however, the difference was negligible and not clinically significant.

## Introduction

Cataract surgery is one of the most frequently performed surgical procedures worldwide [1]. Achieving optimal postoperative refractive outcomes is critical to the success of modern cataract surgery [2]. Despite advancements in preoperative biometry, surgical techniques, and intraocular lens (IOL) technology, refractive surprises remain a persistent challenge, contributing to patient dissatisfaction and suboptimal visual results [3].

Astigmatism is a common refractive error caused by irregular curvature of the cornea or lens. Correction of astigmatism in cataract surgery may be achieved through the implantation of a toric IOL, which incorporates cylindrical power to neutralize corneal astigmatism [4]. While toric IOLs represent a significant advancement in cataract and refractive surgery, residual postoperative astigmatism remains a challenge. The implantation of toric IOLs significantly lowers the amount of residual astigmatism compared to when a non-toric IOL is implanted [5]. However, Hirnschall et al. found that over one-third of patients failed to achieve their targeted refraction following toric IOL implantation [6].

The accuracy of the IOL power formula which a surgeon relies on to select the IOL for a patient is crucial to the postoperative refractive outcome. Many toric IOL formulae are commercially available, and more are in development. Variations in postoperative refractive predictions exist between formulae due to differences in their handling of preoperative biometric and demographic variables. Accurate prediction of the effective lens position (ELP) is crucial, as the ELP significantly impacts the effective cylindrical power of toric IOLs at the corneal plane [7].

The IOLMaster 700 (Carl Zeiss Meditec AG, Jena, Germany) offers the Barrett TK Toric (BTT) and Haigis Toric (HT) formulae directly on its user interface for the calculation of toric IOL power, thus reducing the risk of transcription errors. The BTT formula builds on the Barrett Universal II formula, which uses a theoretical model, paraxial ray tracing, and data-driven refinements to estimate the ELP and IOL power based on thick lens optics [8]. The HT formula uses a linear regression approach and is based on thin lens optics. It uses three constants (a0, a1, and a2) to estimate ELP, incorporating axial length (AL) and anterior chamber depth (ACD) [9]. Although the HT formula may be considered somewhat outdated compared to newer-generation formulae, it remains relevant as one of the toric IOL formulae directly available on the IOLMaster 700.

In clinical practice, the availability of multiple IOL formulae can create uncertainty for surgeons when selecting the most appropriate option for an individual patient. This study aims to evaluate the accuracy of the BTT and HT formulae in predicting postoperative refractive outcomes following toric IOL implantation.

## Materials and methods

Patient population

This retrospective observational study was undertaken at Chatswood Private Hospital and Sydney Adventist Hospital in Sydney, Australia. Medical records were reviewed to identify patients who underwent uncomplicated cataract surgery with the implantation of an AcrySof IQ Toric IOL (models SN6AT3 to SN6AT6; Alcon Laboratories Inc., Fort Worth, Texas, United States) between January 1, 2022, and December 31, 2023. All operations were performed by Dr. Keith Ong, an ophthalmic surgeon, at Chatswood Private Hospital and Sydney Adventist Hospital in Sydney, Australia. Eyes were excluded if the procedure was combined with another ophthalmic procedure, if the procedure was complicated, if the postoperative best corrected visual acuity after one month was less than 20/40, or if there was any concurrent ocular or systemic disease causing significant visual impairment. In patients where both eyes were eligible, the first operated eye was included in the study.

Ethics

The Human Research Ethics Committee of Adventist HealthCare Limited waived the need for ethics approval and the need to obtain consent for the collection, analysis, and publication of the retrospectively obtained and anonymized data for this non-interventional study. The study was conducted in accordance with the tenets of the Declaration of Helsinki.

Surgical technique 

The corneal limbus was marked at 3 o'clock and 9 o'clock positions using a preoperative toric reference marker with the patient in the seated position to prevent cyclotorsion. Cataract phacoemulsification was performed using the Alcon Constellation system (Alcon Laboratories Inc., Fort Worth, Texas, United States) and a 0.9 mm mini-flared 45° Kelman phaco tip through a 2.75 mm corneal incision at the axis of astigmatism. The toric IOL was inserted in the capsular bag, and the IOL was rotated to the final position by aligning reference markings to the desired axis.

Data collection

Demographic data were collected on patient age at the time of surgery, sex, ethnicity, and eye laterality. Preoperative AL, ACD (distance from the corneal epithelium to anterior lens surface), lens thickness (LT), white-to-white distance (WTW), total keratometry (TK), and axis were measured using swept-source optical coherence tomography (SS-OCT) by the IOLMaster 700 (Version 1.90.12.05). The IOLMaster 700 measures both the anterior and posterior corneal curvatures, to determine the total corneal astigmatism, expressed as TK. This differs from standard keratometry which only measures the anterior corneal curvature. TK was recorded in two meridians, the flat meridian (TK1) and the steep meridian (TK2). IOL power and toricity were calculated using the BTT and HT formulae accessed via the IOLMaster 700. Predicted residual postoperative spherical and cylindrical refractive error and astigmatism axis were adjusted to the AcrySof IQ Toric IOL model that was implanted in that eye. Surgically induced astigmatism due to incision was set at 0 D at 0° for all calculations. Subjective refraction was determined postoperatively at least four weeks postoperatively. All assessments and measurements were conducted by the same ophthalmic surgeon. 

Data analysis

Spherical equivalent (SE) refraction was calculated as the sum of the spherical component of refraction added to half the cylindrical component of refraction. SE prediction error (PE) was defined as the difference between the postoperative and the predicted residual SE. Astigmatism PE was calculated as the difference between the postoperative manifest refractive astigmatism corrected for the corneal plane and the predicted residual astigmatism.

Major calculations were performed on Microsoft Excel (Microsoft Corporation, Redmond, Washington, United States). Data analysis was performed using IBM SPSS Statistics for Windows, Version 27.0 (Released 2020; IBM Corp., Armonk, New York, United States). A p-value less than 0.05 was considered statistically significant. Descriptive statistics were applied for demographic data. Sample size calculation was not performed as the data was collected as a convenient sample. The Kolmogorov-Smirnov and Shapiro-Wilk tests were performed to determine the normality of the data. The paired t-test was used to compare means of parametric data. The Wilcoxon signed-rank test was used to compare means of non-parametric data. Cochran's Q test and post-hoc pairwise McNemar tests were conducted to compare the proportions of eyes achieving absolute PE within thresholds (≤0.25 D, ≤0.50 D, ≤0.75 D, ≤1.00 D) between BTT and HT formulae.

Vector analysis was used in calculations regarding astigmatism. The double-angle plot method, as described by Abulafia et al., was used to describe the preoperative corneal astigmatism and postoperative manifest refractive astigmatism at the corneal plane. Double-angle plots were used to demonstrate the mean absolute and centroid postoperative refractive astigmatism PE by vector analysis [10].

## Results

The study included 52 eyes from 52 patients. Table 1 summarizes the demographics and clinical characteristics of the study population.

**Table 1:** Descriptive data Values are presented as mean±SD (range) or n (%). ACD: anterior chamber depth; IOL: intraocular lens; LT: lens thickness; SE: spherical equivalent; TK1: flat meridian; TK2: steep meridian; WTW: white-to-white distance *Cylinder power of the toric IOL at the IOL plane

Study population's demographics and clinical characteristics	
Number of patients	52
Number of eyes	52
Age (years)	72.4±6.2 (61-93)
Sex	
Male	27 (51.9%)
Female	25 (48.1%)
Ethnicity	
East Asian	48 (92.3%)
Caucasian	4 (7.7%)
Eye laterality	
Right	27 (51.9%)
Left	25 (48.1%)
AL (mm)	24.30±1.12 (22.28-27.76)
ACD (mm)	3.04±0.39 (2.34-4.15)
TK1 (D)	43.22±1.34 (40.13-47.04)
TK2 (D)	44.89±1.40 (41.51-48.81)
LT (mm)	4.77±0.32 (4.04-5.42)
WTW (mm)	11.76±0.33 (11.2-12.3)
Implanted IOL sphere power (D)	19.58±3.27 (12.0-27.0)
Implanted IOL cylinder power (D)*	2.25±0.60 (1.50-3.75)
Postoperative SE refraction (D)	-0.96±0.43 (-1.75-0.13)
Preoperative corneal astigmatism	1.67±0.47 (0.97-2.87)
Postoperative refractive astigmatism	0.31±0.37 (0.00-1.50)
Days between operation and postoperative assessment	290±186 (33-747)

SE

Table 2 outlines the comparison of SE PE between the BTT and HT formulae. The median absolute error (MedAE) of BTT was greater than HT, but the difference did not reach statistical significance (0.195 vs. 0.185 D; p=1.000). The mean absolute error (MAE) of both formulae was 0.257 D.

**Table 2:** Spherical equivalent prediction error Means is expressed as mean±SD (range). ME: mean numerical prediction error; MedAE: median absolute error; MAE: mean absolute error

	ME (D)	P-value	MedAE (D)	P-value	MAE (D)
Barrett TK Toric	-0.049±0.327 (-0.85 to 0.89)	0.008	0.195	1.000	0.257±0.206 (0.01-0.89)
Haigis Toric	-0.114±0.328 (-1.15 to 0.50)		0.185		0.257±0.231 (0.01-1.15)

**Figure 1:**
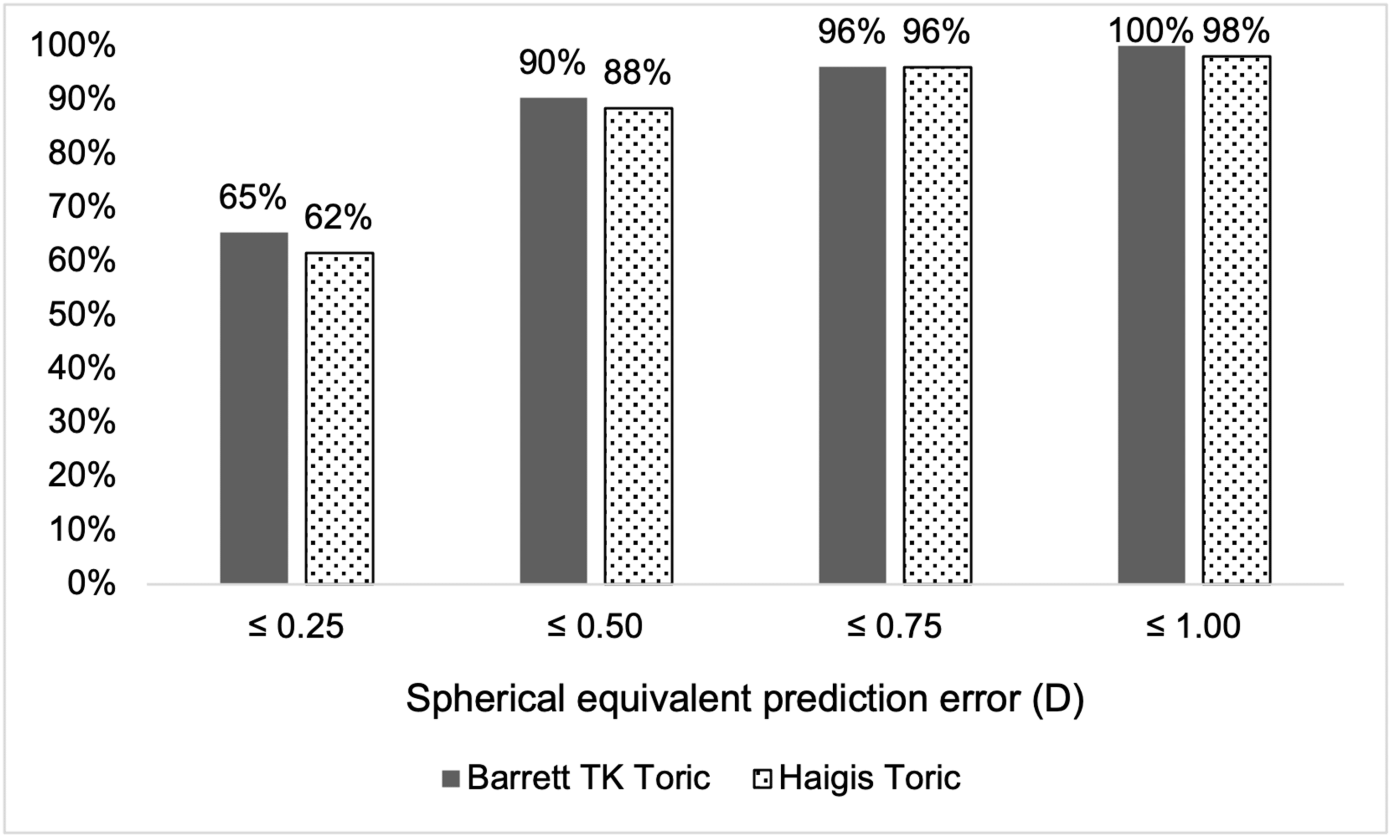
Percentage of eyes with absolute spherical equivalent prediction error within diopter thresholds (n=52)

Figure 1 outlines the percentage of eyes with absolute SE PE within various diopter thresholds. BTT had more eyes within the ≤0.25 D, ≤0.50 D, and ≤1.00 D thresholds and the same number of eyes within the ≤0.75 D threshold compared to HT. Across all groups, there are no significant differences in proportions between BTT and HT (p=0.791-1.000).

Astigmatism

**Figure 2:**
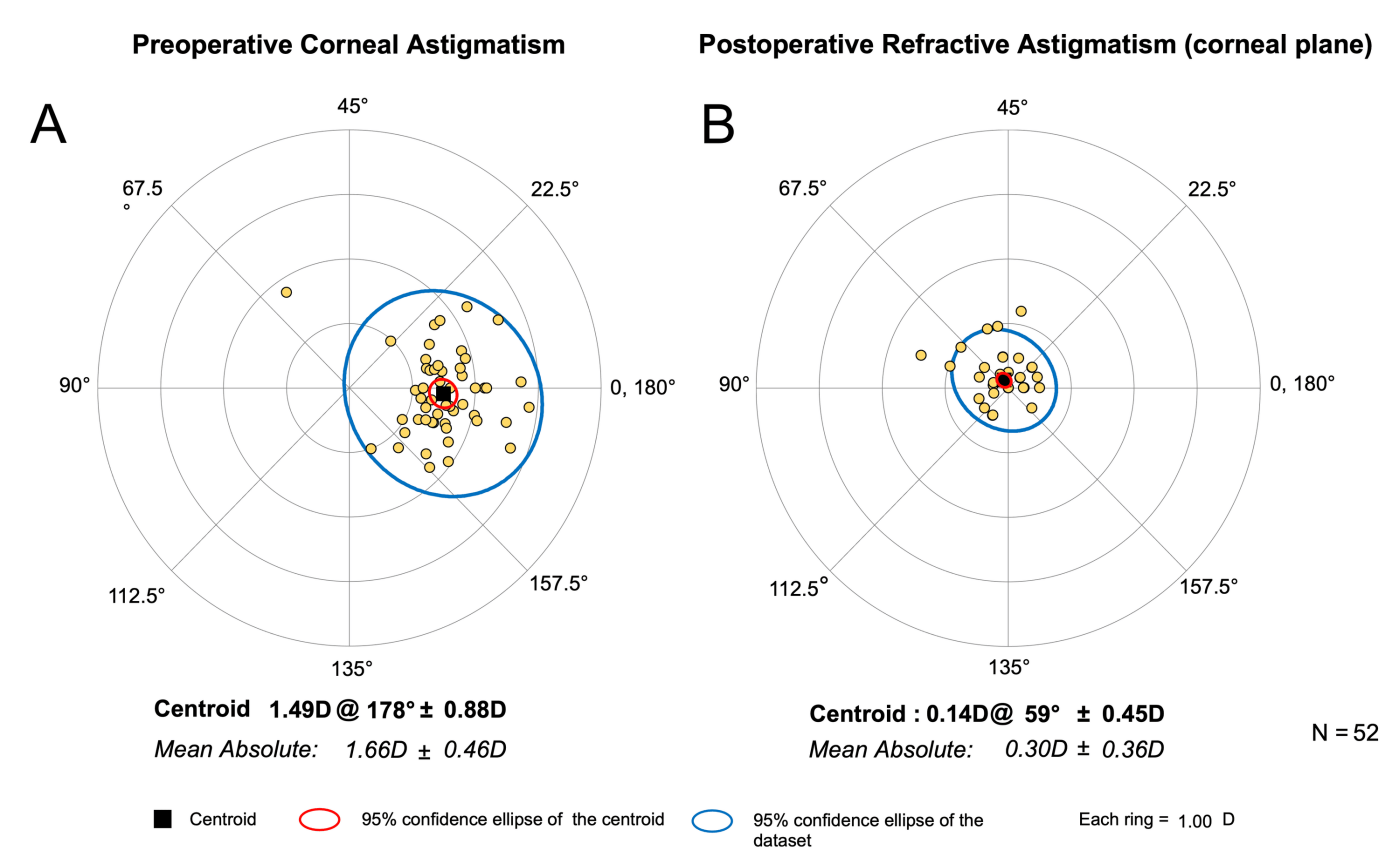
Double-angle plots of the (A) preoperative corneal astigmatism and the (B) postoperative refractive astigmatism at the corneal plane

The centroid of the preoperative corneal astigmatism was 1.49 D @ 178°±0.88 D, and the centroid of the postoperative refractive astigmatism at the corneal plane was 0.14 D @ 59°±0.45 D (Figure 2) for the overall cohort. 

Table 3 outlines the MAE and centroid error in predicted residual astigmatism by the two formulae. HT tended to produce smaller astigmatism PE compared to BTT (MAE 0.41±0.33 D vs. 0.47±0.36 D; p=0.003). The centroid error by HT was less than BTT (0.18 D @ 70°±0.50 D vs. 0.32 D @ 76°±0.50; p<0.001 for x and y components). 

**Table 3:** MAE and centroid error in predicted residual astigmatism, as determined by vector analysis MAE: mean absolute error

	MAE (D)±SD	P-value	MAE range (D)	Mean centroid (D @ angle)±SD	P-value
Barrett TK Toric	0.47±0.36	0.003	0.02-1.77	0.32 @ 76°±0.50	<0.001 (both x and y components)
Haigis Toric	0.41±0.33		0.00-1.67	0.18 @ 70°±0.50	

**Figure 3:**
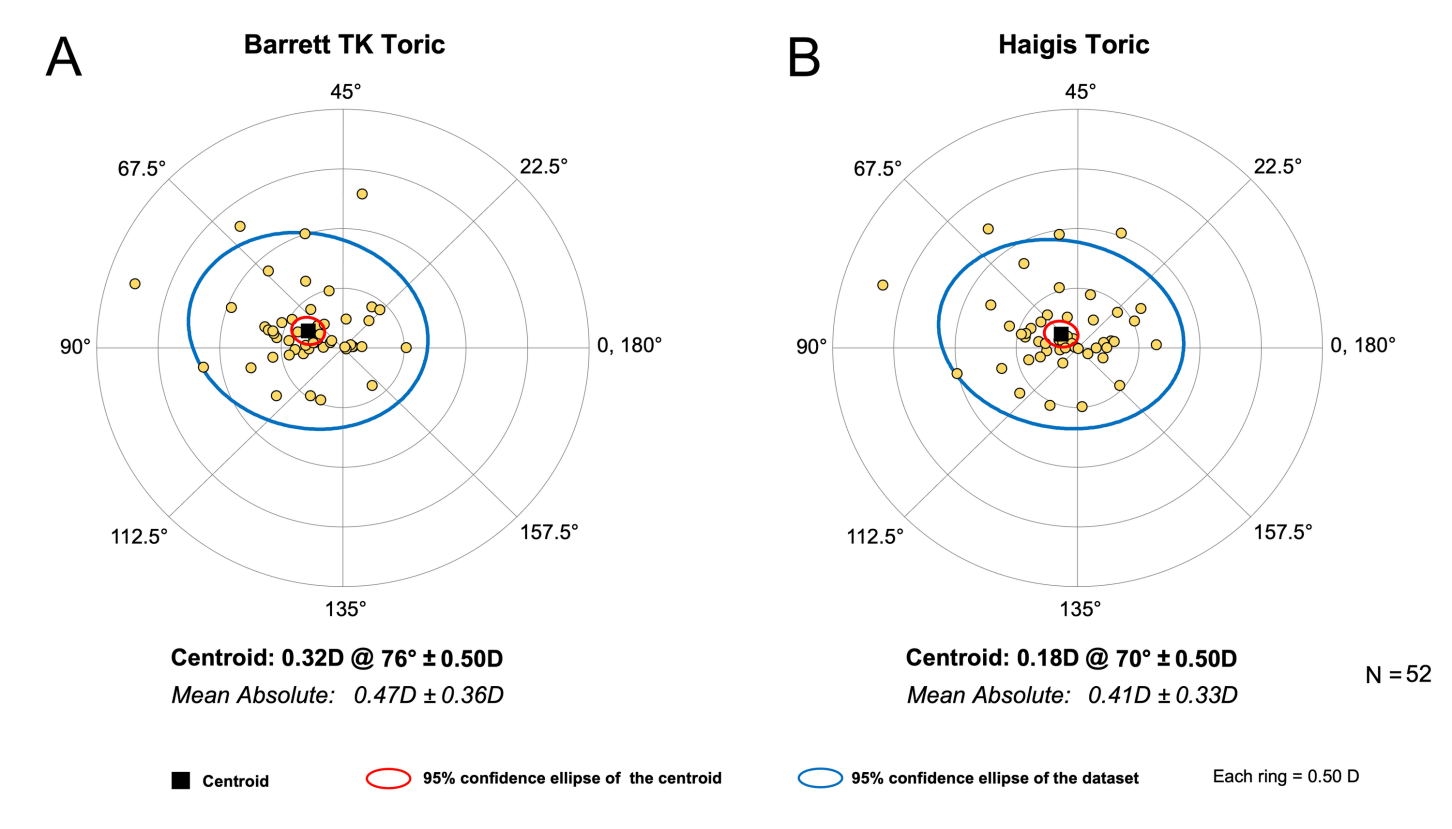
Double-angle plots of the postoperative refractive astigmatism prediction error estimated by the (A) Barrett TK Toric formula and the (B) Haigis Toric formula. Vector analysis was employed to determine the centroid and mean absolute error

Figure 3 shows the double-angle plots of postoperative refractive astigmatism PE of the two formulae. 

**Figure 4:**
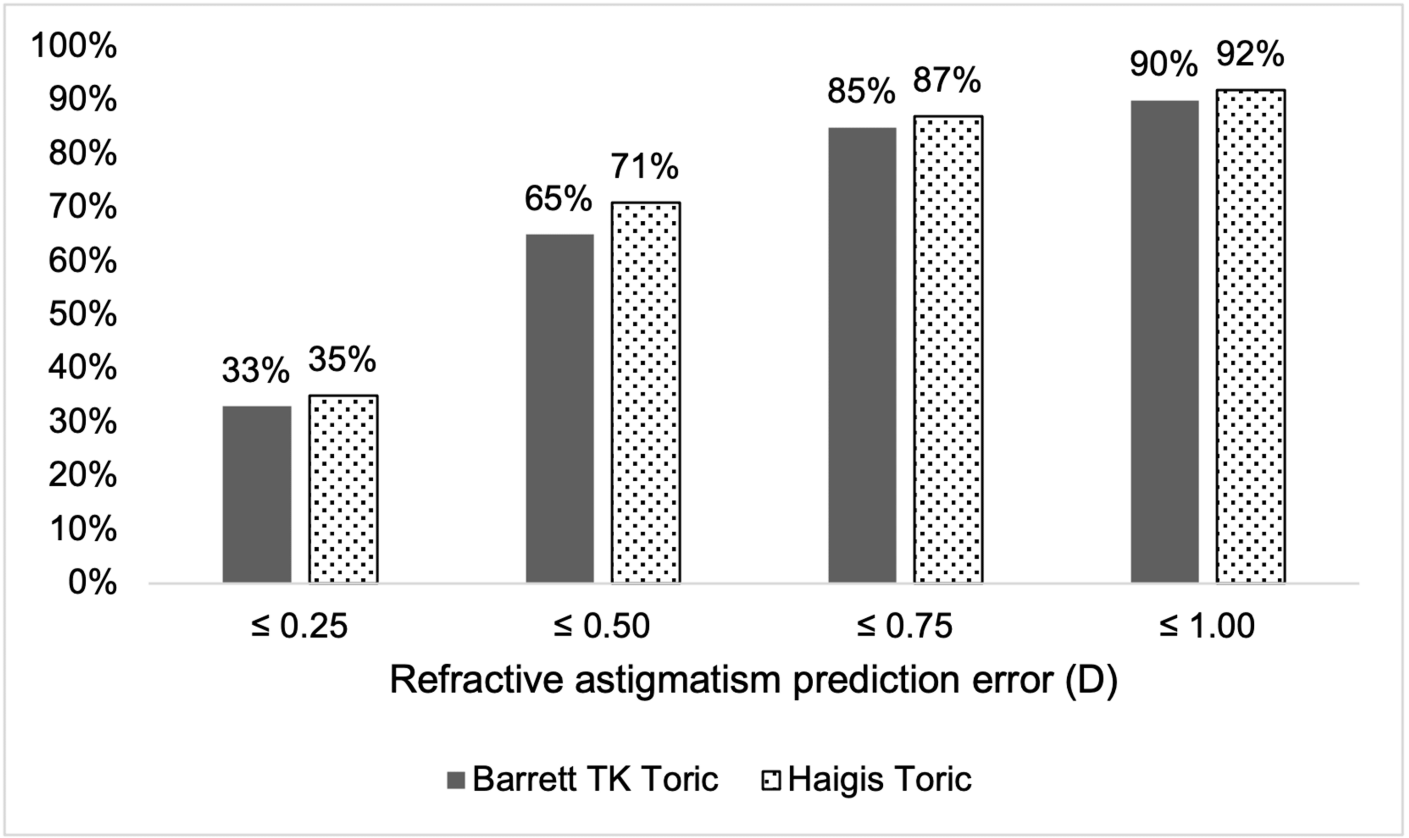
Percentage of eyes with absolute refractive astigmatism prediction error within diopter thresholds (n=52)

Figure 4 outlines the percentage of eyes with absolute refractive astigmatism PE within various diopter thresholds. HT had more eyes than BTT within all thresholds.

## Discussion

The advent of toric IOLs has enhanced refractive correction for patients with pre-existing astigmatism undergoing cataract surgery. Surgeons use IOL power formulae to predict the postoperative refractive outcome, thus enabling them to select the most suitable IOL to meet each patient's needs and preferences. This study assessed the accuracy of the BTT and HT formulae in predicting postoperative spherical equivalent and refractive astigmatism error after the implantation of a toric IOL. 

The advantage of new-generation formulae over older-generation formulae for SE prediction in non-toric IOLs has been well described [11]. However, accuracy in the prediction of non-toric IOLs does not necessarily guarantee the performance in toric IOLs. We found that both the BTT and HT formulae demonstrated similarly high accuracy in predicting postoperative SE for a toric IOL and there was no statistical difference in MedAE or MAE between the two formulae. Our results of 65% and 62% of eyes within ±0.25 D of SE PE for Barrett and Haigis, respectively, are higher than in the study by Shi et al. (51.2% and 45.9%, respectively) [12]. The greater accuracy of our result may be attributed to the use of a single experienced surgeon performing all biometric measurements and surgeries, thus minimizing bias from inter-surgeon variability. The incorporation of TK is shown to improve the prediction of residual refractive astigmatism for toric IOLs [13-15]; however, its role in non-toric IOL prediction accuracy is debated [16,17].

A direct comparison between BTT and HT formulae for astigmatism prediction for toric IOL has not, to the best of our knowledge, been reported previously. In this cohort, we found that HT outperformed the BTT formula in predicting postoperative refractive astigmatism. However, the magnitude in differences is unlikely to be clinically significant. We used the vector analysis method as described by Abulafia et al. [10] which accounts for both the magnitude and axis of astigmatism. The difference in astigmatism MAE between the two formulae was 0.06 D, which is clinically insignificant. The proportion of eyes achieving astigmatism PE within ±0.50 D was 65% and 71% for Barrett TK Toric and Haigis Toric, respectively, in our study. For the Barrett formula, Xian et al. [18] reported 54.7% of eyes, and Kane and Connell [19] reported 53.7% falling within ±0.50 D of astigmatism PE. Eom et al.'s [20] study of a similar sample size reported a higher percentage for the Haigis formula of 75.3% despite a lack of posterior corneal keratometry measurements.

It is well established that the effective toricity of a given toric IOL is dependent on the ELP. Older toric calculators, such as the original Alcon toric online calculator, assumed a fixed ratio between the cylindrical power at the IOL and corneal planes, therefore resulting in undercorrections in long eyes and overcorrections in short eyes [21]. Both the BTT and HT formulae are vergence-based toric formulae, whereby the effective toricity of the toric IOL is based on its spherical power and the predicted ELP. Since the BTT formula considers more biometric variables in its calculations compared to the HT formula, the Barrett formula may have an advantage in estimating ELP and postoperative refractive astigmatism more precisely, but this was not observed in our study.

We observed that the BTT and HT formulae provided different suggestions for the implantation orientation of the toric IOL for the same patient. This may be due to inherent differences in the mathematical models of the two formulae. The BTT formula uses a vergence-based approach with empirical validation to estimate the ELP and predict the toric effect at the corneal plane. The HT formula estimates ELP based on three lens constants (a0, a1, and a2), AL, and ACD, although the theoretical handling of toricity prediction is unclear as it is proprietary to the IOLMaster 700. 

The strengths of this study were that all assessments and surgeries were performed by the same surgeon utilizing the same surgical method, biometry device, and toric IOL lens model, thus minimizing variability and the impact of surgically induced astigmatism. We reported a comprehensive analysis of refractive prediction for spherical equivalence and astigmatism which are both relevant to the general ophthalmologist in clinical practice. Our study was designed with a "real-world" approach for greater applicability of the results to the general ophthalmologist in clinical practice. Although the use of postoperative keratometry values and postoperative IOL placement axis would eliminate the effect of surgically induced astigmatism and postoperative IOL rotation [19], both these factors are obviously unknown to the surgeon in the preoperative planning phase. By utilizing the power predictions generated by the IOLMaster 700, the error resulting from transcribing data to an external third-party or online calculator was eliminated. Other modern toric IOL formulae were not included in this study to ensure this study was relevant to surgeons who wish only to select between formulae available on the IOLMaster 700. Furthermore, the inclusion of formulae from online calculators may introduce bias from transcription errors from the manual input of biometry data. Eyes with the best corrected visual acuity of less than 20/40 after one month were excluded as per the protocol suggested by Hoffer and Savini [22].

The limitations of this study included the relatively small sample size, which precluded subgroup analyses for factors known to influence IOL power calculations, such as AL [23], ACD [12,23], and eyes with with-the-rule and against-the-rule astigmatism [24]. Sample size calculation was not performed because sampling was performed by convenience. We acknowledge that the lack of sample size calculation may impact statistical power. Postoperative IOL axis misalignment can be a source of error in refractive predictions, but this factor was not assessed in this study. In this study, surgically induced astigmatism was assumed to be 0 D for all patients in order to improve the generalizability of the results. We do, however, acknowledge that real-world variability exists and therefore reflects a limitation of this study. A source of bias to consider is error in biometry measurements which can be affected by inadequate eye opening [25]. A significant majority of this cohort was of East Asian descent, which tend to have narrower palpebral apertures compared to people of Caucasian descent [26].

## Conclusions

This study compared the accuracy of the BTT and HT formulae in predicting postoperative SE and astigmatism refractive error after toric IOL implantation. Both formulae demonstrated high and comparable accuracy in predicting postoperative SE outcomes. While the HT formula showed statistically better performance in astigmatism prediction, the difference was negligible and not clinically significant. We also found that the two formulae sometimes suggested different toric IOL implantation orientations for the same patient, possibly due to variations in their mathematical models. Future studies with larger cohorts are needed to validate these findings and explore formula performance across a broader spectrum of eyes.
